# Alternative Test Models: Toxicity Testing Takes Stock

**DOI:** 10.1289/ehp.116-a113a

**Published:** 2008-03

**Authors:** Bob Weinhold

Early February 2008 saw the celebration of the first 10 years of work by the Interagency Coordinating Committee on the Validation of Alternative Methods (ICCVAM), the federally funded body charged with promoting the regulatory acceptance of scientifically valid safety testing methods that replace, reduce, or refine the use of animals. ICCVAM also released a five-year plan establishing priorities for research, translation, and validation activities. The plan was unveiled during an anniversary symposium held 5 February 2008 in Bethesda, Maryland.

ICCVAM conducts technical evaluations of alternative testing methods proposed for regulatory use, makes recommendations to regulatory agencies on the usefulness and limitations of new methods, identifies knowledge and data gaps that need to be addressed with further research and development efforts, and coordinates with similar efforts internationally. The National Toxicology Program Interagency Center for the Evaluation of Alternative Toxicological Methods (NICEATM), housed at the NIEHS, provides administrative, operational, and scientific support to the inter-agency committee.

To date, ICCVAM’s recommendations have resulted in national and international acceptance of alternatives for acute oral toxicity, skin corrosivity, and allergic contact dermatitis, three of the most common types of toxicity assays. ICCVAM has also recommended use of the Bovine Corneal Opacity and Permeability and Isolated Chicken Eye tests to assess eye irritation; regulatory decisions on these assays are due in April 2008.

William Stokes, director of NICEATM, says the tests reviewed by ICCVAM are essential to translating research findings from bench to bedside. “Our ‘bedside’ is more effective public health prevention measures,” he says. “The improved alternatives recommended by ICCVAM help prevent disease and injury so you don’t end up in the bed in the first place.”

“ICCVAM is essential,” says John Bailey, executive vice president for science at the Personal Care Products Council (formerly the Cosmetics, Toiletry, and Fragrance Association). “It brings the science together, and allows a transparent assessment.”

ICCVAM’s new five-year plan lists four areas of emphasis: establishing priorities for future testing; identifying and encouraging appropriate research efforts; educating stakeholders about the acceptance and appropriate use of improved methods; and improving partnerships with and between those inside and outside ICCVAM. Some of the approaches emphasized for further development include high-throughput assays, use of species such as roundworms and tadpoles, and better biomarkers of toxic effects.

Despite the committee’s successes, ICCVAM’s efforts have received mixed reviews. Jessica Sandler, director of the Regulatory Testing Division for People for the Ethical Treatment of Animals, who strongly supported ICC-VAM when it was formed, says progress has been painfully slow. She attributes this in part to ICCVAM not taking advantage of the work of sister organizations such as the European Centre for the Validation of Alternative Methods (ECVAM), which has endorsed the use of many more alternative test methods than its U.S. counterpart.

But Stokes explains that ICCVAM, NICEATM, and ECVAM in fact worked very closely in recent years. ICCVAM’s activities are limited to the review of test methods applicable to regulatory testing, while ECVAM has a broader mandate to address animal use for all areas of research and testing (e.g., screening to prioritize chemicals for product development). Stokes notes that ECVAM is a center with numerous laboratories and a large full-time staff, whereas ICCVAM is a committee with no laboratories. ICCVAM must also operate with a high level of transparency and the opportunity for broad public and stakeholder input in contrast to ECVAM’s less-extensive closed review process. Moreover, Stokes says, many of the assays recommended by ECVAM have not been accepted by European regulatory agencies.

Daniel Krewski, chairman of a National Research Council committee that produced the 12 June 2007 report *Toxicity Testing in the 21st Century*, affirms the value of ICCVAM’s work so far. However, the committee recommends that an entirely different federal framework, possibly with a size and budget akin to that of the National Toxicology Program, be rapidly adopted to overcome ICCVAM’s limitations. Such a program would be “quite a paradigm shift from what we’re doing now,” he says, but ICCVAM could be viewed as a transitional step toward this new direction.

Meanwhile, the new five-year plan may help expedite improvements, says Sonya Lunder, a senior analyst with the Environmental Working Group. She has many concerns about the ICCVAM review process but appreciates the plan: “It gives us a benchmark to measure progress.” The plan is available at http://iccvam.niehs.nih.gov/docs/5yearplan.htm.

